# Supplementary material for: Species-Specific Flash Patterns Track the Nocturnal Behavior of Sympatric Taiwanese Fireflies
Source: Biology (Basel). 2022 Jan 1;11(1):58. doi: 10.3390/biology11010058 (PMC8773436; doi:10.3390/biology11010058)
Supplement: Supplementary file 1 [file biology-11-00058-s001.zip › biology-1497730-Supplementary/biology-1497730-supplementary.pdf]

biology

MDPI

Supplementary Materials: Species-Specific Flash Patterns Track the Nocturnal Behavior of Sympatric Taiwanese Fireflies

King-Siang Goh [1], Chia-Ming Lee [2] and Tzi-Yuan Wang [3,*]

[1] Genomics Research Center, Academia Sinica, Nankang, Taipei, 115, Taiwan; gohks0505@gmail.com or gohks@gate.sinica.edu.tw
[2] Research Center for Applied Sciences, Academia Sinica, Nankang, Taipei, 115, Taiwan; charlee@gmail.com or charm@gate.sinica.edu.tw
[3] Biodiversity Research Center, Academia Sinica, Nankang, Taipei, 115, Taiwan
* Correspondence: tziyuan@gmail.com or sds@gate.sinica.edu.tw; Tel.: +886-2-2787-2258

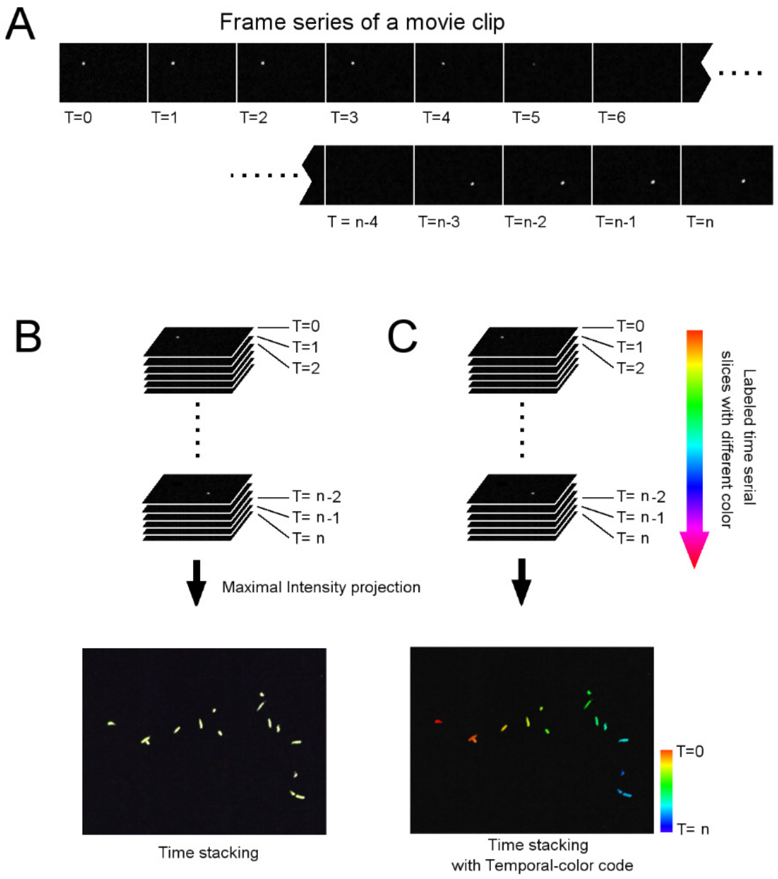

Figure S1. Time-stacking method on the firefly flash signal tracking. A) The flash signaling in the consecutive frames of a movie clip is a constantly shifting, disappearing and reappearing light spot along with time passing (T=0, T=1….T=n). Superimposing and projecting the consecutive frames reveals a time-stacking image showing B) the flash trajectory (lower left panels) or C) chronological order (corresponding to the color code) of the flashes (lower right panels). The conversion of consecutive frames of a movie clips to a time-stacking image has been well described in Materials and Methods.

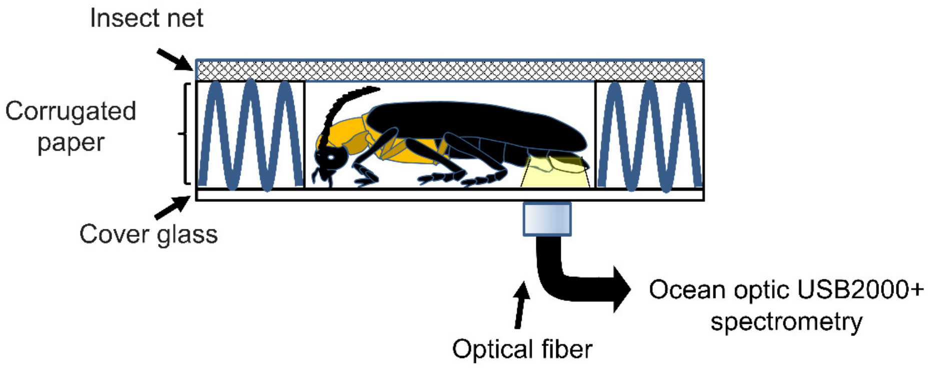

Figure S2. A homemade firefly holder for measuring flash wavelength. The measurements were performed by immobilizing individual fireflies in the hollow chamber (1x1.5x0.4 cm^3) of a home-made holder. The holder consists of an upper steel insect-net (mesh pore size ~0.1 cm), a middle corrugated paper (5x5x0.4 cm^3), and a lower cover glass (2.2x2.2x.0.017 cm^3). The coreless endcap of the optical fiber connecting to the spectrometry is placed below the light organ for collecting the wavelength.

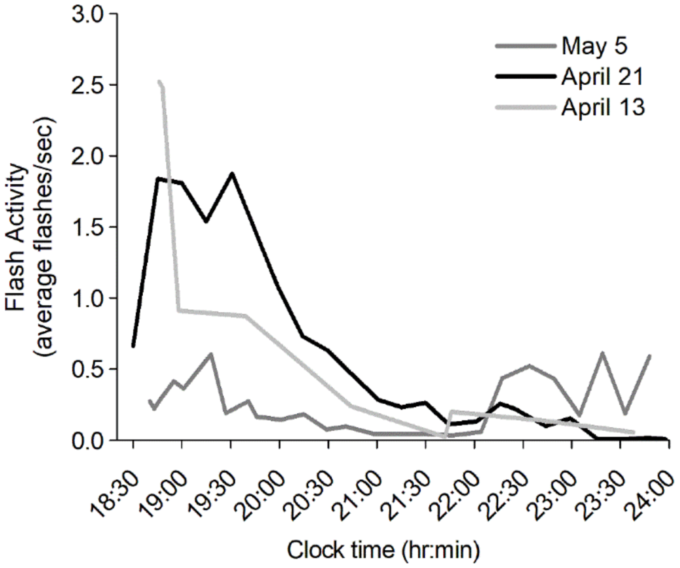

Figure S3. Nightly change in population flash activities during *A. cerata* mating season along the Genliao hiking trail. The time-course chart denotes the change in population flash activities (total flashes/s) at 18:30–24:00 on 13 April (light gray line), 21 April (black line), and 5 May (dark gray line), 2018. The flash signals were recorded every 15–60 min using a fixed-point digital camera, shot at a frame rate of 30 fps and stored as 1–2 min video clips. Flash activities (average flashes/s) was evaluated by the automated flash spot detection as described in Materials and Methods.

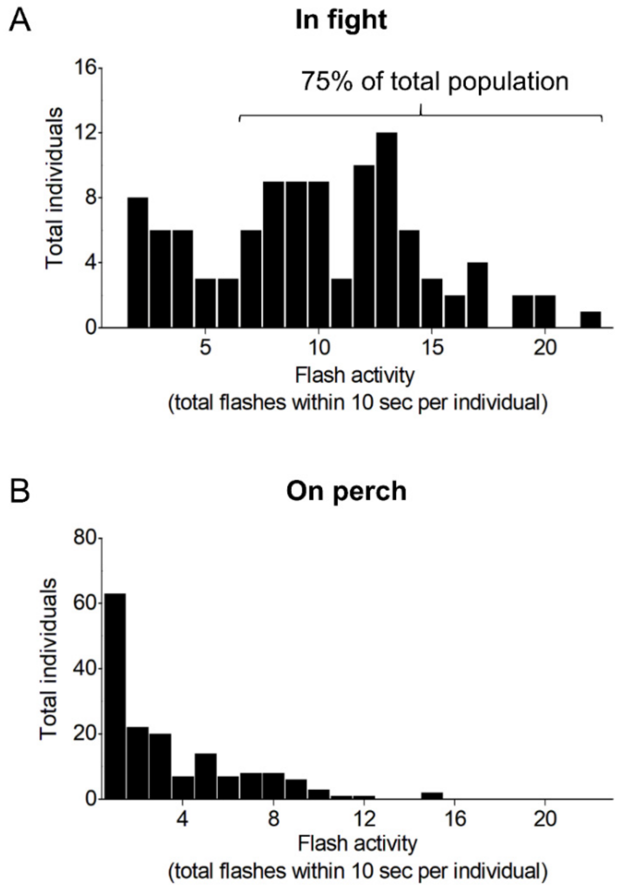

Figure S4. Statistic of total flashes emitted by each individual in the case study population. The distribution of total numbers of fireflies A) in flight and B) on perch that emit 1–22 flashes per 10 s are shown. A total of 104 flying individuals and 161 perching individuals from the case study population were analyzed. The individuals that emitted ≥7 flashes per 10 s, comprising 75% of the total flying population, were further identified of their species using FI pattern matching. These individuals and their flashes were determined by the manually spatiotemporal tracking of the flash signals in 19 of the video clips recorded on 21 April 2018.

Video S1. An example video file of handheld tracking of a flying male *A. cerata* individual in flashing recorded with a digital camera at Genliao hiking trail (Taipei). The video show the single-pulse flash pattern with a low frequency (~1.2 flashes/s) and yellowish flash color of an individual while patrolling flight. (MP4, 18.2MB)

Video S2. An example video file of handheld tracking of a flying male *L. kagiana* individual in flashing recorded with a digital camera at Genliao hiking trail (Taipei). The video show the triple-pulse flash pattern with a middle frequency (~2.2 flashes/s) and orange-yellowish flash color of an individual in patrolling flight. (MP4, 33.4MB)

Video S3. An example video file of handheld tracking of a flying male *L. curtithorax* individual in flashing recorded with a digital camera at Genliao hiking trail (Taipei). The video show the dense single-pulse flash with a high frequency (~4.4 flashes/s) and orange-yellowish flash color of an individual in flight. (MP4, 37.1MB)
