# Supplementary figures and images for: Species-Specific Flash Patterns Track the Nocturnal Behavior of Sympatric Taiwanese Fireflies
Source: Biology (Basel). 2022 Jan 1;11(1):58. doi: 10.3390/biology11010058 (PMC8773436; doi:10.3390/biology11010058)

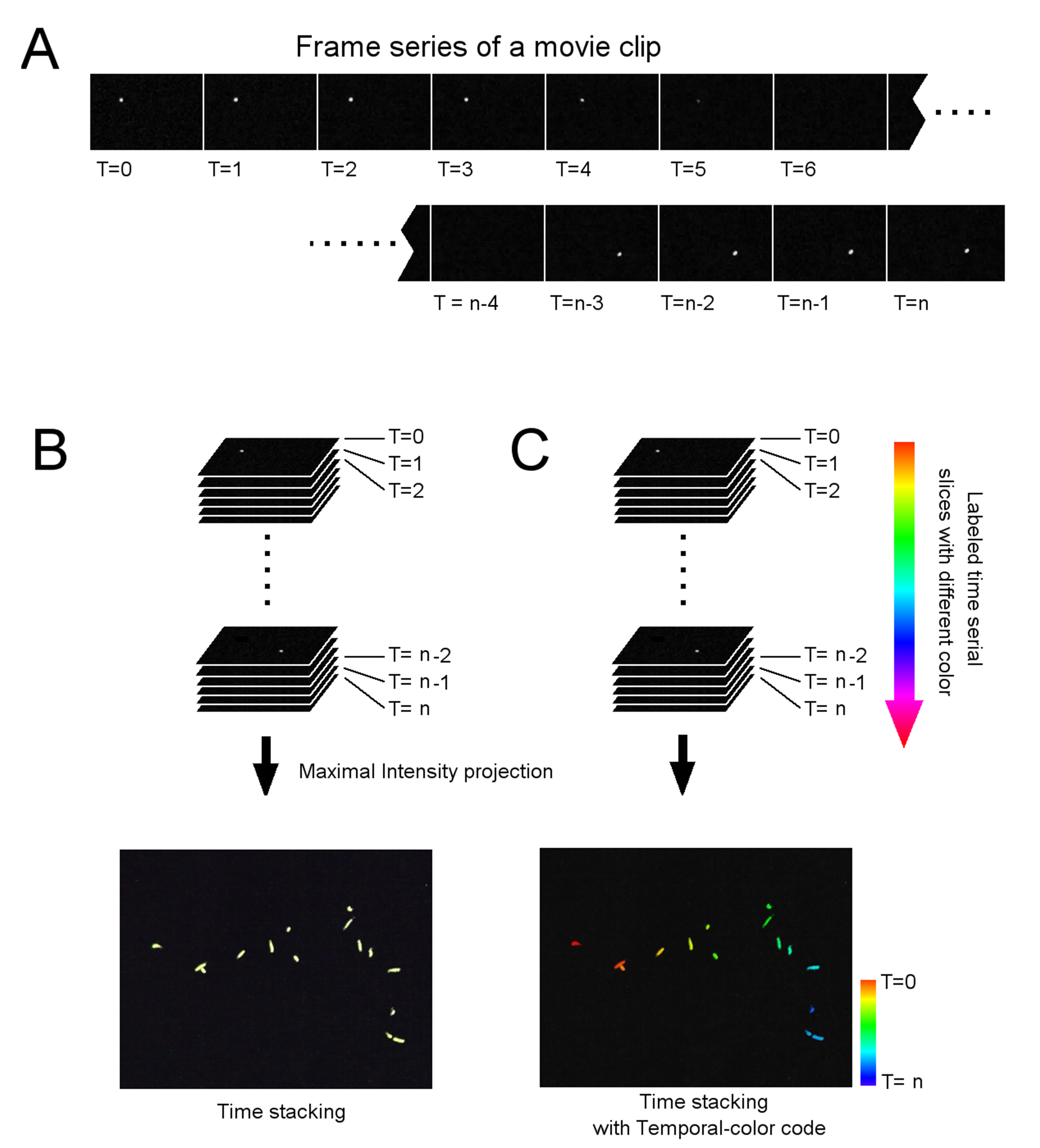

Supplement: Supplementary file 1 [file biology-11-00058-s001.zip › biology-1497730-Supplementary/Figure S1.tif]

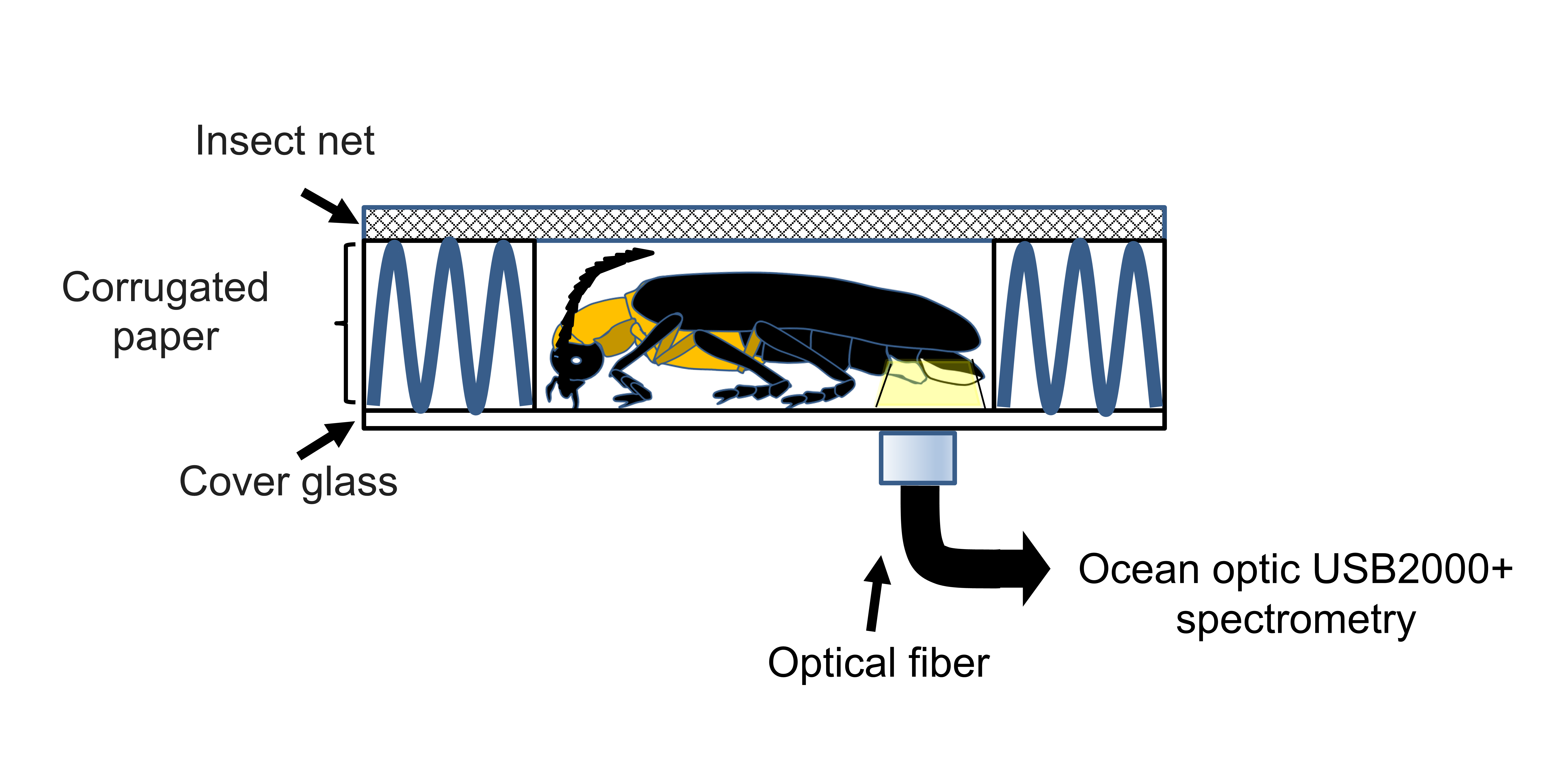

Supplement: Supplementary file 1 [file biology-11-00058-s001.zip › biology-1497730-Supplementary/Figure S2.tif]

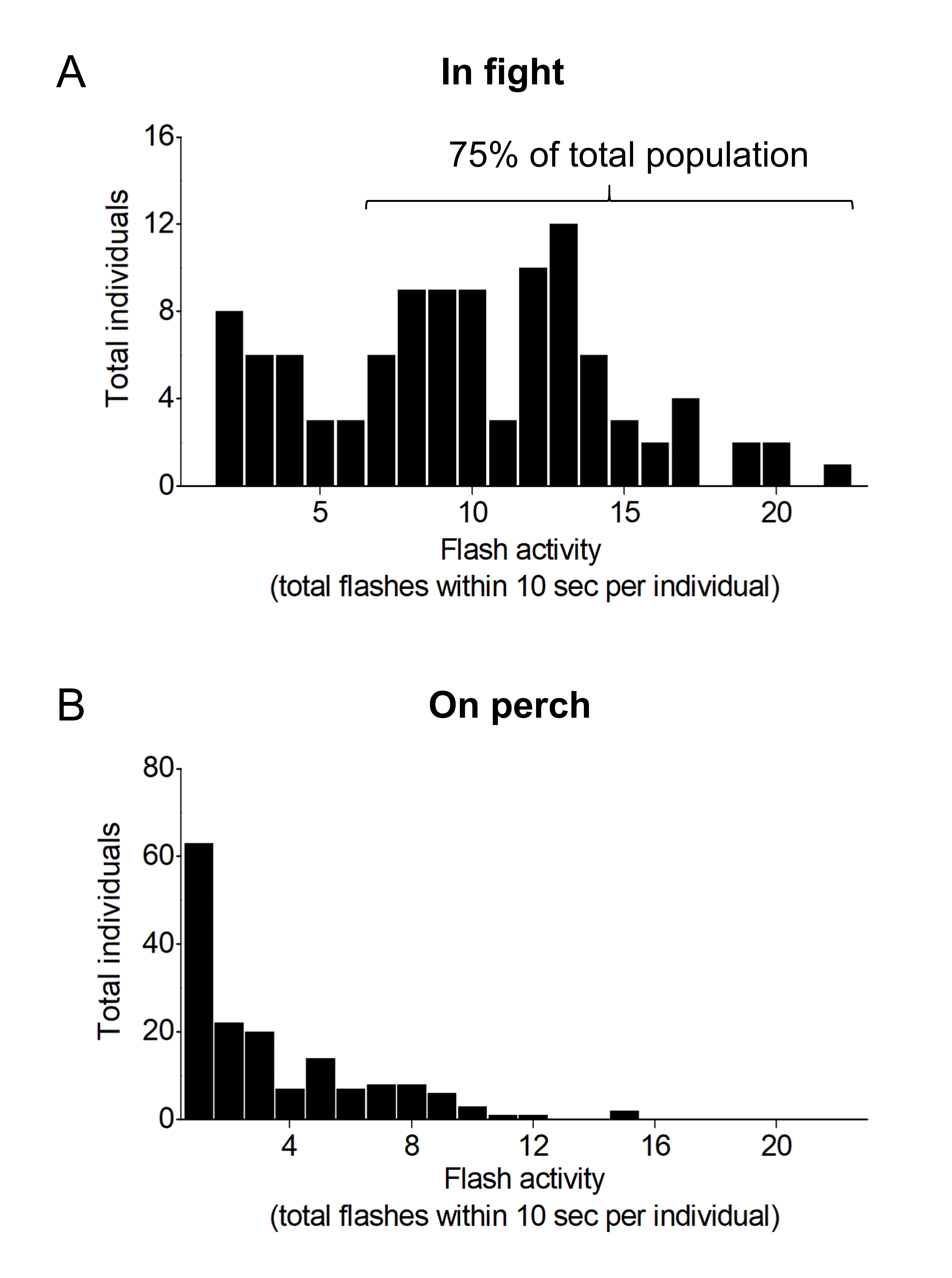

Supplement: Supplementary file 1 [file biology-11-00058-s001.zip › biology-1497730-Supplementary/Figure S4.tif]
